# Supplementary material for: Risk factors analysis of 90-day mortality in patients with sepsis in intensive care unit
Source: PLoS One. 2025 Jun 20;20(6):e0325813. doi: 10.1371/journal.pone.0325813 (PMC12180717; doi:10.1371/journal.pone.0325813)
Supplement: Attachment — Submitted filename: Response to Reviewers.docx [file pone.0325813.s001.docx]

**Subject: Clarification Regarding Ethical Approval and Request for Resubmission Opportunity**

Dear Editor,

Thank you very much for your detailed feedback regarding our manuscript titled *"****Risk Factors Analysis of 90-Day Mortality in Patients with Sepsis in the Intensive Care Unit.****"* I truly appreciate the time and effort the editorial team has dedicated to reviewing our submission.

I would like to take this opportunity to clarify the points raised in your letter. Contrary to the concerns expressed in your email, the necessary ethical approval for the study was indeed obtained prior to accessing the data. Unfortunately, due to an oversight on our part, the exact dates of the study period were not clearly stated in the manuscript, which I understand caused some confusion. This was not an intentional omission but rather a misunderstanding regarding the specific formatting of the dates. I sincerely apologize for this error.

In response to your request, we have now revised the manuscript to include the exact dates when the data were accessed for research purposes, formatted as DD/MM/YYYY, as required. We believe these changes now fully comply with the submission guidelines and have made all necessary adjustments accordingly.

We have carefully addressed the comments provided by the reviewers and the academic editor in the previous decision letter (PONE-D-25-04707). In particular, we have now included the exact study dates and have ensured all other requested clarifications are incorporated into this new version.

We are more than willing to revise the manuscript to include any additional information needed to fully comply with your ethical approval requirements. Given this clarification and the revisions made, I would be grateful if you could kindly consider allowing us to resubmit the manuscript as a revised version of our previous submission (PONE-D-25-04707).

Thank you once again for your time and understanding. I look forward to your response and hope that we can resolve this matter in a manner that meets the standards of *PLOS ONE.*

Sincerely,
Dr. Yuan
hfyy2020@126.com
