# Supplementary material for: Risk factors analysis of 90-day mortality in patients with sepsis in intensive care unit
Source: PLoS One. 2025 Jun 20;20(6):e0325813. doi: 10.1371/journal.pone.0325813 (PMC12180717; doi:10.1371/journal.pone.0325813)
Supplement: Attachment — Submitted filename: Response_to_Reviewers_Revised.docx.docx [file pone.0325813.s002.docx]

Dear Editor and Reviewers,

We sincerely thank you for your thoughtful review and valuable comments on our manuscript entitled "Risk factors analysis of 90-day mortality in patients with sepsis in intensive care unit" (Manuscript ID: PONE-D-25-08186). We appreciate the opportunity to revise our manuscript and have carefully addressed each comment. Below is a detailed point-by-point response.

**Reviewer #1**

Comment 1: Nice study but no novelty.

Response: Thank you for your comment. Although several studies have explored the relationship between body composition and mortality in critically ill patients, few have focused specifically on sepsis patients using abdominal CT scans, particularly within the Chinese population. Our study adds value by evaluating CT-derived skeletal muscle parameters (SMD, SMA, SMI) in relation to 90-day mortality in sepsis patients, using robust multivariable models. We have emphasized this aspect in the revised Introduction and Discussion sections.

Comment 2: How many patients were screened in the study period? And how many were excluded? Please add patients flow.

Response: Thank you for the suggestion. We have added a patient flow diagram (Figure 3) that details the screening process, exclusion criteria, and final sample size.

Comment 3: How did you identify sepsis? Do you have data set of that?

Response: We have clarified this in the Methods section. Sepsis was defined according to the Sepsis-3 criteria as infection with an increase in SOFA score ≥2. Diagnosis was based on clinical judgment supported by laboratory and imaging findings; microbiological confirmation was not required.

Comment 4: How is GLIM criteria, if possible.

Response: We appreciate this point. Unfortunately, due to the retrospective nature of our study, the required components for GLIM criteria (e.g., weight change, food intake, muscle mass by non-imaging methods) were not consistently available in the records. This limitation has been acknowledged in the Discussion section.

Comment 5: How is the functional aspect, because mortality is already reported.

Response: We agree that functional status is an important prognostic factor. However, performance scores or functional assessments were not routinely documented in the medical records, and thus could not be included. This has been explained as a study limitation.

Comment 6: Please clarify multivariate analysis more clearly. I cannot understand which factors are included.

Response: We have revised the Statistical Analysis section to clearly define the models:

- Unadjusted model: body composition variable only.
- Model 2: adjusted for SOFA score, age, and sex.
- Model 3: adjusted for SOFA score, age, sex, and CCI. These models are now explicitly described in both the Methods section and table legends.

Comment 7: Age and sex should be included at least in the supplemental file. Same result?

Response: Thank you. Age and sex have been included in the multivariable models (Model 2 and Model 3), and results remained significant. This is now clearly presented in the Results section and tables.

Comment 8: Either of SOFA or APACHE should be chosen, not both.

Response: We agree. We retained SOFA as the adjustment variable in all multivariable models due to its direct link to the Sepsis-3 definition. APACHE II was used only in descriptive analyses. This choice is now clarified in the Methods and Discussion sections.

Comment 9: Please emphasize the novelty of this research.

Response: We have added text in the Introduction and Discussion to emphasize that this study is among the first to evaluate CT-derived skeletal muscle parameters in relation to 90-day mortality in sepsis patients in a Chinese ICU cohort, using multiple adjusted models.

**Reviewer #2**

Major Comment 1: Would similar results be observed for 30-day or in-hospital mortality?

Response: Thank you for this valuable suggestion. Unfortunately, due to the retrospective design and incomplete follow-up data, 30-day and in-hospital mortality were not available for analysis. This limitation has been acknowledged in both the Methods and Discussion sections.

Major Comment 2: Is adjustment for CCI (Charlson Comorbidity Index) not necessary?

Response: We agree and have now included CCI in Model 3 to assess its potential confounding effect. The results remained statistically significant, suggesting that skeletal muscle parameters are independent predictors of mortality even after adjusting for comorbidities. This is described in the revised Methods, Results, and Discussion sections.

Minor Comment: "Patients were excluded..." should be placed in the "Patient and data collection" section.

Response: Thank you. We have moved the relevant exclusion criteria description from the CT analysis section to the Patient and Data Collection section as suggested.

Table 2, 3 Comment: "Model 2" is not defined.

Response: We have now explicitly defined all models in the Statistical Analysis section and included explanatory footnotes in Tables 2 and 3.

We hope that the revisions made adequately address all reviewer concerns. We sincerely thank the reviewers and editors for their constructive feedback, which has helped improve the quality of our manuscript.

Sincerely,

Dr. Yuan, on behalf of all authors
