# Supplementary material for: Risk factors analysis of 90-day mortality in patients with sepsis in intensive care unit
Source: PLoS One. 2025 Jun 20;20(6):e0325813. doi: 10.1371/journal.pone.0325813 (PMC12180717; doi:10.1371/journal.pone.0325813)
Supplement: Attachment — Submitted filename: Response_to_reviewers_auresp_2.docx [file pone.0325813.s003.docx]

**Manuscript ID: PONE-D-25-08186R1**

**Title: Risk factors analysis of 90-day mortality in patients with sepsis in intensive care unit**

We thank the Academic Editor and the reviewers for their valuable time and constructive feedback. We carefully addressed each of the comments, and all changes have been incorporated into the revised manuscript. Below is our point-by-point response. **Reviewer #1**

Comment:

All comments have been addressed.

Response:

We sincerely thank the reviewer for the positive evaluation and endorsement of our revised manuscript.

**Reviewer #2**

Major Comment:

The authors mention that mortality data were collected retrospectively and via follow-up calls. However, it is unclear whether in-hospital mortality was extracted from institutional records such as the electronic medical record. Clarification would be helpful, especially given that 90-day but not 30-day or in-hospital mortality is reported.
In addition, clearly stating the data sources for other key variables in the study would further enhance the transparency and credibility of the research.

Response:

Thank you for this important suggestion. We have now clarified the data sources in the Patients and Data Collection section of the Methods. Specifically, in-hospital mortality and other clinical variables (including age, sex, BMI, SOFA and APACHE II scores, laboratory results, CCI, and NUTRIC scores) were obtained from the hospital’s electronic medical record system. Post-discharge survival within 90 days was obtained through structured telephone follow-up.

Furthermore, as noted in the Limitations section of the manuscript, we did not analyze 30-day or in-hospital mortality due to incomplete follow-up data and variation in hospital length of stay—common limitations in retrospective datasets. We also explained that while 30-day mortality is often used as a short-term outcome indicator, 90-day mortality is a widely accepted endpoint that better reflects long-term outcomes in critically ill patients.

Minor Comment:

It would be helpful if the figures are numbered according to the order in which they are first mentioned in the text.

Response:

Thank you for pointing this out. We have carefully revised the manuscript and reordered figure numbers to align with the sequence in which they are first referenced in the main text.

**Additional Notes:**

- The data underlying this study have been deposited in Figshare and are publicly accessible: https://doi.org/10.6084/m9.figshare.28873667

- We used the PACE figure tool to ensure compliance with PLOS ONE's figure formatting requirements.

- We confirm that no retracted articles are cited in the reference list.

We appreciate the opportunity to revise our manuscript, and we believe the current version has been significantly improved. Thank you again for your consideration.

Sincerely,

Dr. Yuan (Corresponding Author)

On behalf of all co-authors
